# Gaze perception from head and pupil rotations in 2D and 3D: Typical development and the impact of autism spectrum disorder

**DOI:** 10.1371/journal.pone.0275281

**Published:** 2022-10-27

**Authors:** Diana Mihalache, Peter Sokol-Hessner, Huanghao Feng, Farzaneh Askari, Nuri Reyes, Eric J. Moody, Mohammad H. Mahoor, Timothy D. Sweeny

**Affiliations:** 1 Department of Psychology, University of Denver, Denver, Colorado, United States of America; 2 Changshu Institute of Technology, Jiangsu, China; 3 Department of Electrical and Computer Engineering, McGill University, Montréal, Québec, Canada; 4 Department of Pediatrics, JFK Partners, University of Colorado Anschutz Medical Campus, Aurora, Colorado, United States of America; 5 Wyoming Institute for Disabilities, University of Wyoming, Laramie, Wyoming, United States of America; 6 Department of Electrical & Computer Engineering, University of Denver, Denver, Colorado, United States of America; Istituto di Fisiologia Clinica Consiglio Nazionale delle Ricerche, ITALY

## Abstract

The study of gaze perception has largely focused on a single cue (the eyes) in two-dimensional settings. While this literature suggests that 2D gaze perception is shaped by atypical development, as in Autism Spectrum Disorder (ASD), gaze perception is in reality contextually-sensitive, perceived as an emergent feature conveyed by the rotation of the pupils and head. We examined gaze perception in this integrative context, across development, among children and adolescents developing typically or with ASD with both 2D and 3D stimuli. We found that both groups utilized head and pupil rotations to judge gaze on a 2D face. But when evaluating the gaze of a physically-present, 3D robot, the same ASD observers used eye cues less than their typically-developing peers. This demonstrates that emergent gaze perception is a slowly developing process that is surprisingly intact, albeit weakened in ASD, and illustrates how new technology can bridge visual and clinical science.

## Introduction

The ability to discriminate another person’s direction of gaze is critical to human interaction [1, 2]. It reveals direction of attention or goals [3], promotes language development [4], and contributes to theory of mind [1]. Impairment in gaze perception is a key diagnostic symptom in Autism Spectrum Disorder (ASD) [5]. Accordingly, referential gaze—the direction of gaze toward an object in space—has been studied extensively in individuals with ASD [6], with an initial emphasis on eye cues. Yet in order to perceive gaze direction, the visual system relies on information from *both* the eyes and head [7, 8]. Accordingly, recent examinations have explored additive effects of head and eye cues on the development of joint attention, showing that redundant cueing from coarse variations in these features increases both gaze following [9, 10] and the amount of time spent looking at a target of joint attention [10, 11]. Yet surprisingly little is known about how typically-developing (TD) children and children with ASD *integrate* fine and sometimes conflicting gaze cues to resolve another person’s precise direction of attention. Here, we investigated how children and adolescents with ASD and typically-developing peers combine information from head and pupil rotations to perceive gaze in 2D and 3D.

**Fig 1:**
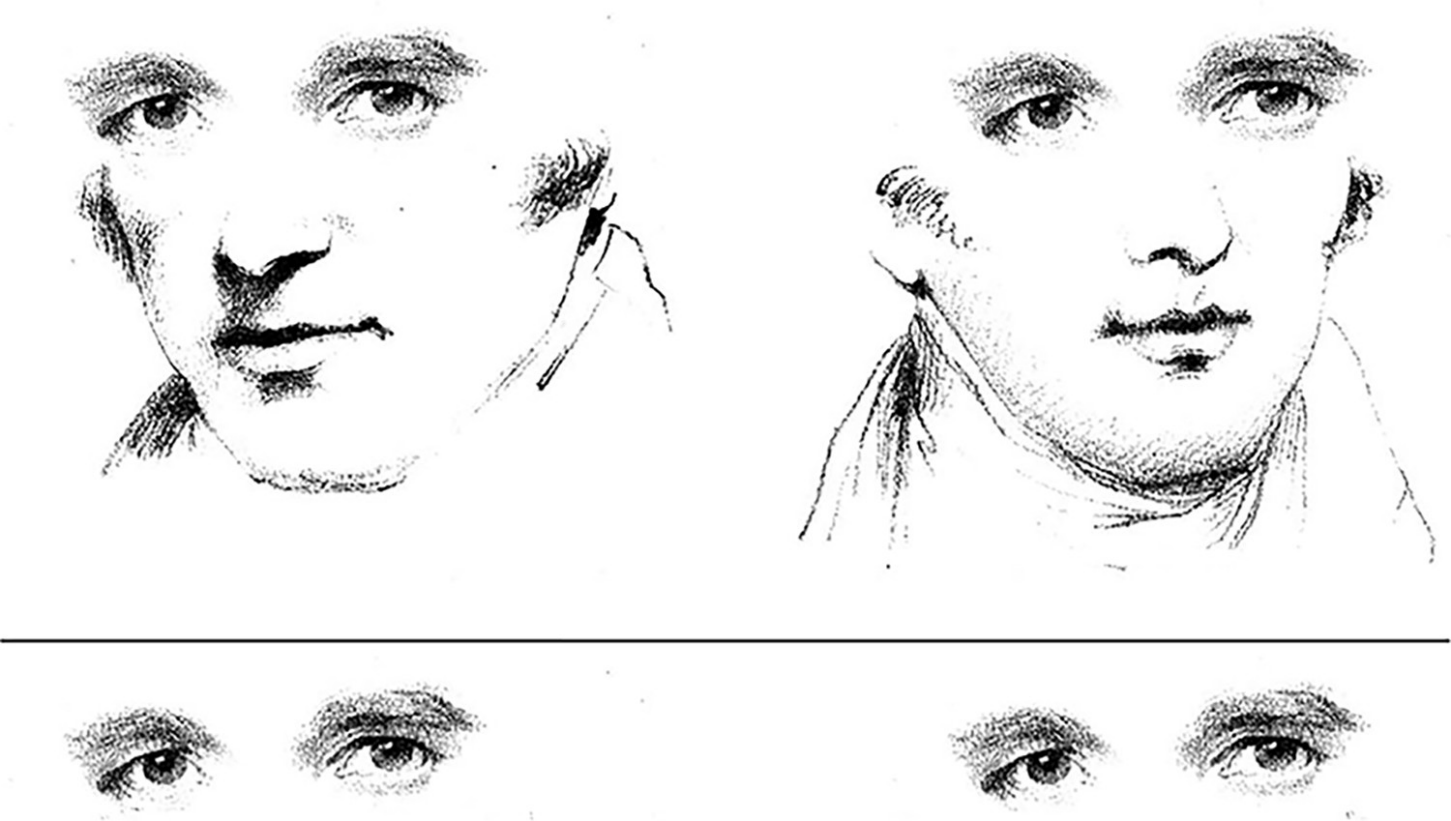
Eyes with identical pupil rotations appear to have unique gaze directions when coupled with leftward or rightward head rotations [8].

Evidence that gaze is perceived in an integrated way, as an emergent feature, is evident in the architecture of the visual system and from perceptual experience itself. For example, neurons in the superior-temporal sulcus selectively code not just for eye and head rotations, but also combinations of both cues [12]. Perceptually, the rotations of the head and eyes are fused into a singular, holistic experience of gaze direction (Fig 1), whereby the orientation of the head attracts [13, 14] or repels [15] the perceived rotation of the eyes. Although a few researchers have suggested that head and eye cues may be weighted differently among individuals or special populations [7, 16], to our knowledge this hypothesis has received very little attention. This is surprising since individuals with ASD are known to struggle with holistic visual processing [17, 18].

Individuals with ASD show impairments in gaze processing compared to their typically-developing peers, including slower processing of direct gaze [19], lower accuracy at discriminating gaze direction [20], and fewer joint attention behaviors [21]. Interactive gaze behaviors are also predictive of ASD. For example, 10-month old infants who were later diagnosed with ASD were shown to initiate joint-attention less frequently than infants who did not receive a subsequent diagnosis of ASD [10]. In a similar study, infants who were later diagnosed with ASD responded to joint attention from an experimenter differently than their typically developing peers, specifically when a target object was not at the location of the experimenter’s gaze [11]. Young adults with ASD also tend to fixate more on the mouth and less on the eyes than controls, due to deprioritized significance of the eyes [22] and/or increased negative physiological response [23]. These deficits, however, are not universal or consistent amongst individuals with ASD. Other studies have shown accurate processing of averted gaze [6] and intact attentional cueing [24]. In general, there appears to be more support for excess fixation on the mouth and diminished attention on the eyes among adults, but not children, with ASD [25]. Similarly, studies with adults report atypical orienting [26], whereas many children with ASD show intact reflexive shifts of attention [24] and intact gaze behavior toward social stimuli [27]. In addition to chronological age, cognitive abilities may contribute to differences in these findings. There is a wide spectrum of functioning in individuals with ASD, and children with ASD who show comparable cognitive abilities tend to process eye and head directions similarly to their typically-developing peers [28, 29].

These studies suggest that there may be a complex developmental trajectory to gaze perception. Therefore, understanding individual differences in addition to group differences between individuals with or without ASD is important, especially as it pertains to the emergent gaze approach described above [30, 31]. In our previous work we provided a framework for evaluating emergent gaze from 2D faces [7]. We found that, similar to adults, typically-developing children integrated information from both head and pupil rotations to estimate gaze direction. We also found preliminary evidence that children with ASD may use pupil information less than typically-developing children. This study provided the foundation for a more rigorous look at emergent gaze in this population, particularly outside the context of static 2D faces, and with a new, more comprehensively validated sample of ASD participants.

Although 2D and 3D faces contain some redundant perceptual information, there are additional cues present in 3D that could influence perception of gaze. For example, 3D faces provide binocular depth cues, and observers perform poorly on some face perception tasks when 3D information is not available [32]. Most importantly for perception of emergent gaze, rotating the aperture of the eyes on a 3D face tends to *repel* perceived gaze while changing the shape of the head *attracts* perceived gaze [33]. These distinct influences from the head and eyes work in parallel to oppose one another during perception of gaze in 3D faces [15, 34]. Even beyond optical and perceptual considerations, judgments about social cues including gaze and social attention [35, 36] take on additional significance, and even recruit distinct neural networks, when they are made in more embedded contexts in which the perceiver is an interactor rather than a spectator [37]. For these reasons, it is unclear whether knowledge about evaluations of 2D faces should generalize to 3D faces.

Here, for the first time, we examined whether children and adolescents with or without ASD integrate information from head and pupil rotations when evaluating gaze on both 2D and 3D faces. We predicted that as a group, individuals with ASD would rely less on pupils than their typically-developing peers, but with significant individual differences in cue usage across both groups, and possibly across 2D and 3D contexts.

## Materials and methods

### Observers

Twenty-five children and adolescents with ASD (19 male, 6 female; Age *M* = 10.7; SD = 2.58) and 26 typically-developing (TD) children and adolescents (17 male, 9 female; Age *M* = 10.5; *SD* = 2.21) participated in this study. Their legal guardians provided informed consent and the children and adolescents provided informed assent. We recruited observers between 7 and 15 years of age in order to measure emergent gaze perception during a sensitive developmental window; spatial perception develops up to the age of seven [38], perception of global features develops into adulthood [39], and processing of eye gaze develops at least until the age of 11 [16]. Observers and families were recruited through flyers at schools, hospitals, and community centers, research listservs, and word of mouth. Our final sample size (N = 51) was similar to a previous investigation [7] that used similar stimuli, design, and analyses. Unlike in the previous study, this time we evaluated cognitive abilities as well as ASD symptoms, which helped us more thoroughly characterize our sample as well as gather additional information for interpretation of results. Note that our task design included 264–288 trials per observer (see below and Supplemental Materials for details) for a total of 14,664 datapoints. Thus, our sample size is most completely represented by the number of observers combined with the number of datapoints. In addition, our hierarchical analyses consider each data point in the context of all other observers and data points to maximize signal and attenuate the influence of statistical noise. All data and analyses are publicly available online (https://osf.io/rhg4f/).

All experimental protocols were approved by the University of Denver IRB, and the research was carried out in accordance with the provisions of the World Medical Association Declaration of Helsinki. Each observer was accompanied by a legal guardian on two visits to the University of Denver campus. We conducted cognitive and ASD assessments on the first visit to evaluate whether observers could be included in the study, and we conducted the gaze perception experiments on the second visit.

#### Assessment

All observers were administered the Autism Diagnostic Observation Schedule, Second Edition (ADOS-2) [40] and the Wechsler Intelligence Scale for Children, Fifth Edition (WISC-V) [41], to assess symptoms of ASD and cognitive skills, respectively. The ADOS-2 and the WISC-V were administered by a master’s-level graduate student, who was research reliable on the ADOS-2. Observers who had a professional clinical or research evaluation completed within three years prior to starting the experiment were not required to have these measures re-administered (we used scores from eight previous WISC-V evaluations and ten previous ADOS-2 evaluations). We report means, standard deviations, and ranges for our assessment data separately for the TD and ASD group in the Supplementary Materials (S1 Table in S1 File). Nine additional observers were not included in the final data set because they only completed part of the experiment before leaving prematurely or not returning for follow-up appointments. Two additional observers were omitted from the data set because the robot experienced a technical issue during their visit. Another two who were recruited for the ASD sample were later excluded from our study because they did not meet the cut-off criteria on the ADOS-2 during our administration. After data had been collected, and before analysis, three additional observers were excluded based on the experimenter’s qualitative assessment that they experienced behavioral or comprehension challenges that interfered with their ability to participate in the computer and robot tasks. No further exclusions were made, and our final sample thus included only observers who met the criteria for inclusion in the TD or ASD samples who were cooperative and appeared to understand the instructions.

### 2D (Computer) gaze task

#### Stimuli

**Fig 2:**
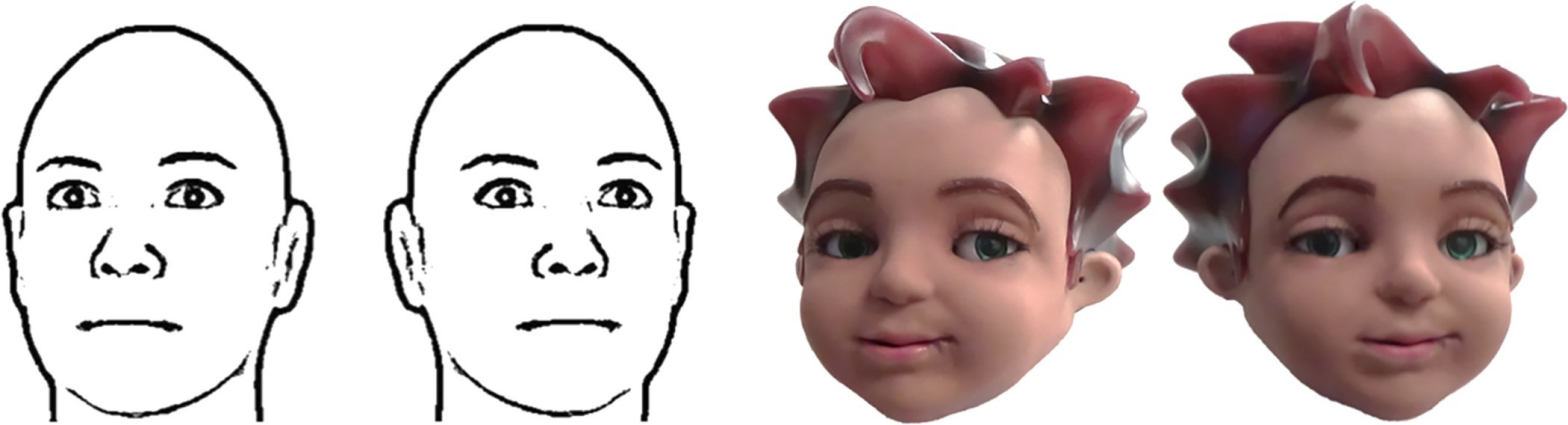
(A) Examples of stimuli from the 2D task and (B) pictures of Zeno from the 3D task. The 2D faces have identical eyes with +5° rotations superimposed on heads with -8° and +8° rotations. The faces from the 3D task have different combinations of head and eye rotations, but both look at the same point in space. The robot face on the left has a head rotation of -8° and an eye rotation of +13°. The robot face on the right has a head rotation of +8° and an eye rotation of -3°.

Our 2D gaze task featured a set of 12 computer-generated faces that we initially created for an investigation with adults [13] and then used subsequently with children [7]. This face set is unique and was designed specifically to measure the strength of a visual phenomenon known as the Wollaston illusion [8]. In this 2D illusion, a pair of eyes looking straight ahead can be made to appear to look leftward when seen in the context of a leftward turned head, or rightward in the context of a rightward turned head [8, 15]. The 2D head subtended a visual angle of 3.76° x 4.76°. The 2D head’s eyes subtended a visual angle of 0.6° x 0.3°. In our 2D face set, the head and pupil cues varied independently, but the rotation, size, and shape of the eye apertures remained fixed (Fig 2A). Specifically, heads had leftward, direct, or rightward rotations (-8°, 0°, and +8°). Pupils had one of four shifts (-25%, -5%, +5%, +25%) within eye apertures from a head with a direct (0°) rotation. These values reflect the percentage, and not the degrees, of a pupil’s shift within the eye opening of a 3D head. The 20% steps between pupil shifts reflected ~5.6° of angular rotation. Using this unique 2D face set allowed us to isolate and measure the pure effect of attraction from head rotation on perceived gaze without an ongoing repulsive effect from changing eye apertures. These stimuli also allowed us to (1) isolate basic processes that contribute to emergent gaze perception, and (2) connect with our recent work in which we used these same 2D stimuli with typically-developing children and adults, and children with a previous diagnosis of ASD [7].

#### Procedure

Each observer was seated 57-cm in front of a 17” laptop screen and encouraged to hold still. On each trial, observers viewed a single face from the 2D stimulus set and indicated whether it appeared to be looking to their (the observer’s) left or right. Each face appeared at the center of the screen against a white background and remained until the observer responded through one of the following methods: (a) pressing left- or right-arrow keys on the laptop keyboard, (b) pointing to their left or right, or (c) saying “left” or “right.” A white screen appeared after their response and remained until the experimenter initiated the next trial by pressing the spacebar. The experimenter did not provide feedback on accuracy, but for the first few trials did offer encouraging words, such as “you’re doing great” and “thank you for working so hard.” Observers completed three blocks of trials for a total of 144 trials with one exception (an observer who completed 120 trials). Each block consisted of the 12 faces from the stimulus set repeated four times; the order of the faces across each block was randomized for each observer.

### 3D (robot) gaze protocol

#### Stimuli/Apparatus

Our three-dimensional gaze task featured combinations of head and pupil rotations displayed on a socially-assistive robot known as Zeno, a 17” tall humanoid research platform from Hanson Robokind (Fig 2B). Zeno’s design includes many tactile and dynamic features, including synthetic skin, neck and facial muscles controlled by 10 motors, and most importantly for this investigation, independent movements of the head and eyes including saccades and blinks, and control of pan and tilt by from independent eye motors. Our team built an API within a program that comes with Zeno (RoboWorkship; RW) to create animations and speech that could be simultaneously executed for rudimentary social interactions with our observers, and to control eye and head movements to specific locations in space. Zeno’s script included saying hello, introducing itself, and providing encouraging words on the first few trials that paralleled the administrator’s words in the 2D task. Zeno’s head was 9 x 10.5cm. Each of Zeno’s eyes were 2 x 0.8cm. The distance between the centers of Zeno’s eyes was 4.8cm. At the viewing distance of 50cm, Zeno’s head subtended a visual angle of 10.2° x 11.8°. Each of Zeno’s eyes subtended a visual angle of 2.29° x 0.91°. Compared to the faces in our 2D set, the 3D robot face provided a more realistic source of gaze because it included additional cues like depth, shading, and convergence of the eyes. We used the same head directions from the 2D task (-8°, 0°, +8°), but we combined these heads with specific pupil directions so that gaze was directed to actual points in space 25° and 5° to the observer’s left, and 5° and 25° to the observer’s right (along a virtual horopter at the distance of the observer from the robot). For example, a -8° 3D head combined with -17° 3D pupils produced a -25° gaze. The eyes and head thus looked at unique points in space in the 3D task. This and other differences from the 2D stimuli (e.g., size, the interactive nature of the robot) made direct comparison between the 2D and 3D tasks challenging. We thus consider the 2D and 3D tasks as two mostly distinct examinations of gaze perception throughout our investigation, and we analyzed data from them separately. For direct 2D/3D comparison analyses, see the Supplementary Materials.

#### Procedure

Each observer was seated in front of Zeno, who was positioned on a table so that its eyes were at approximately the level of the observers’. The experimenter was careful to position Zeno exactly 50-cm from the front of the observer’s face, and to line Zeno up directly in front of the observer at the beginning of the task. As in the 2D task, observers were encouraged to hold still, but because of concerns regarding physical contact, we did not restrain observers (e.g., in a chin rest). Thus, observers were able to move during the task. So long as observers moved randomly throughout the task, this should have only added noise to our estimates of 3D gaze sensitivity, but systematic movement in one direction would have introduced a bias in rightward versus leftward gaze estimates. We note, however, that we were interested in how changes in head and pupil rotations influence gaze judgments, and these effects can be measured independent of stable biases.

On each trial, Zeno produced one of the 12 combinations of head and pupil rotations from the 3D set, and the observer indicated whether Zeno appeared to be looking to their (the observer’s) left or right. Zeno’s gaze remained static until the observer responded by pointing to their left or right or saying “left” or “right.” An experimenter, who was always seated in the testing room to the observer’s left, initiated the next trial by pressing a key on a computer keyboard. The experimenter did not provide any feedback. The robot made statements similar to the ones used in the Computer Task, but no feedback regarding accuracy was provided. Each observer completed six blocks of trials for a total of 144 trials. Each block consisted of the 12 faces from the stimulus set repeated twice; the order of the faces across each block was randomized for each observer. The robot and computer tasks were administered during the same testing session, with the computer task administered first. We initially planned to counterbalance the order of the tasks but decided instead to administer the computer task first in order to obtain appropriate motivation and sustained effort (completing the robot task first could have reduced motivation for the subsequent computer task). It is unclear how running the computer task second might have affected our results. For example, practice effects could have improved performance, whereas boredom might have degraded performance, or the two could have balanced each other out.

#### Analytic approach

Our analytic approach leveraged multiple distinct analyses of observers’ judgments of gaze in the two conditions of the study, enabling us to identify convergent evidence of the usage of pupil and head cues in two- and three-dimensional gaze perception. These included simple averages of judgments, assessments of judgment/cue agreement, and hierarchical generalized linear regressions using the logistic link function that simultaneously accounted for the influence of multiple cues on judgments of gaze while pooling data across all our participants using a hierarchical structure that allows and constrains individual differences. Finally, when performing statistical tests in group-level analyses, we used a combination of parametric and statistically conservative non-parametric tests out of an abundance of caution.

There were some differences between the 2D- and 3D-faces beyond the addition of stereoscopic depth cues. Most notably, the faces in the 2D condition were artificially controlled such that the shapes of the eye apertures did not change as the heads rotated, consistent with prior research [13–15], allowing us to capture a visual illusion often evaluated in examinations of 2D gaze, including our own recent work [7]. In contrast, this feature was necessarily uncontrolled in the case of the 3D faces. We thus considered the 2D and 3D tasks as two mostly distinct examinations of gaze perception throughout our investigation, and we analyzed data from these conditions separately. However, we do acknowledge the merit of contrasting performance across these two tasks from an exploratory standpoint, especially considering the within-subjects nature of our dataset. While suggesting caution in the interpretation of such comparisons, we thus included some direct comparison of cue usage in the two tasks in the Supplementary Materials, and we briefly comment on this relationship in the Discussion along with a reminder about some of the limitations of our design.

## Results

### Preliminary analysis

**Fig 3:**
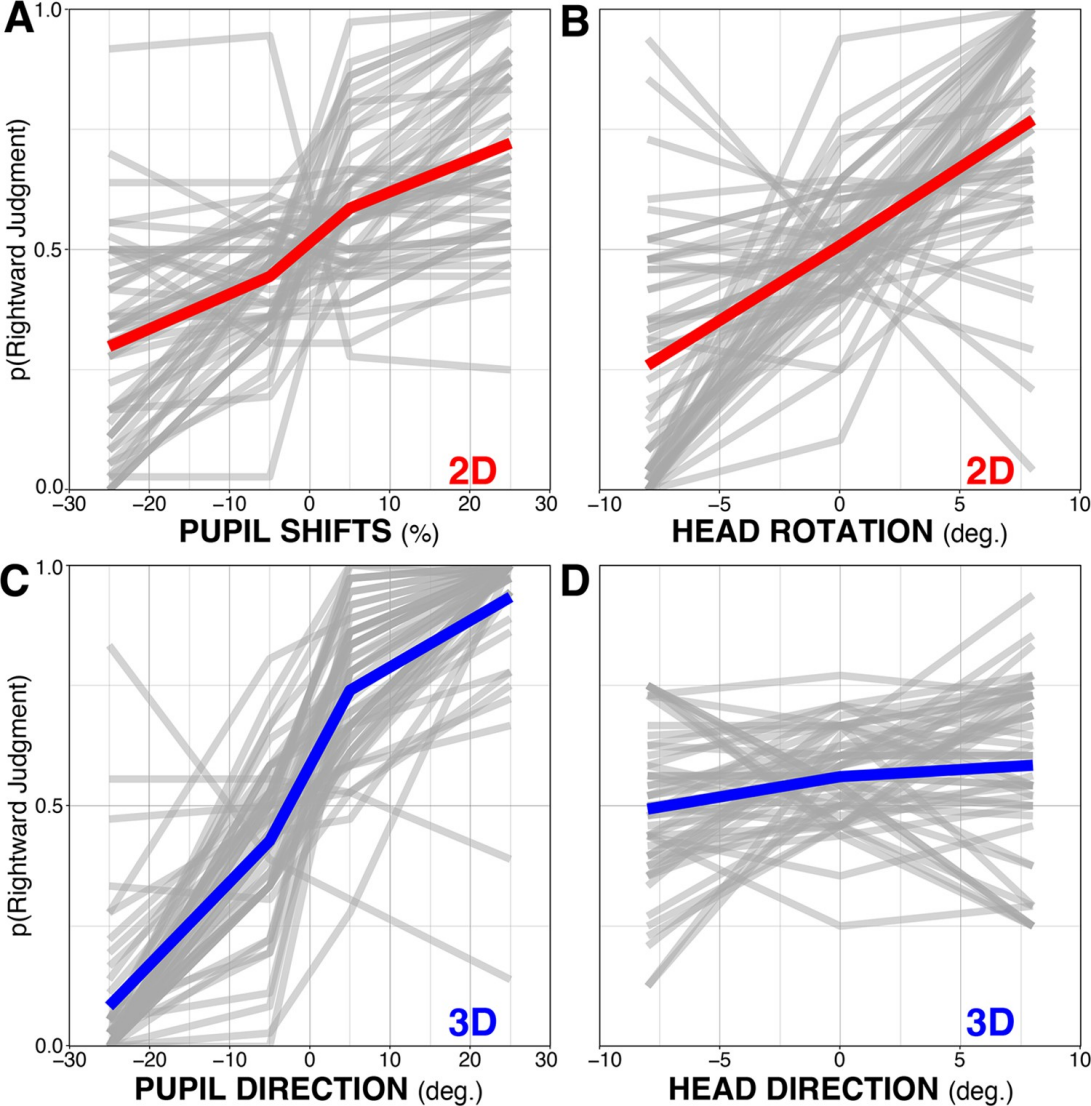
Proportion of rightward judgments as a function of pupil shift (A) and head rotation (B) in the 2D task (red), and pupil direction (C) and head direction (D) in the 3D task (blue). The bold lines represent group-level means.

We began by visualizing the data using simple averaging. For each observer, we calculated the probability that they reported a rightward gaze for each of the four pupil cues, disregarding the different head rotations. We did this separately for data from the 2D and 3D conditions, shown in panels A and C of Fig 3. We then did the same with head cues (disregarding pupil cues) in panels B and D. Several patterns are evident from visual inspection. First, pupil cues mattered in both 2D and 3D conditions—the more rightward the pupils were oriented, the more likely observers were to say “rightward,” but the effect appeared relatively more pronounced in the 3D task. Second, while head rotations were related to “rightward” judgments in the 2D task, observers relied relatively less on head direction in the 3D task.

**Fig 4:**
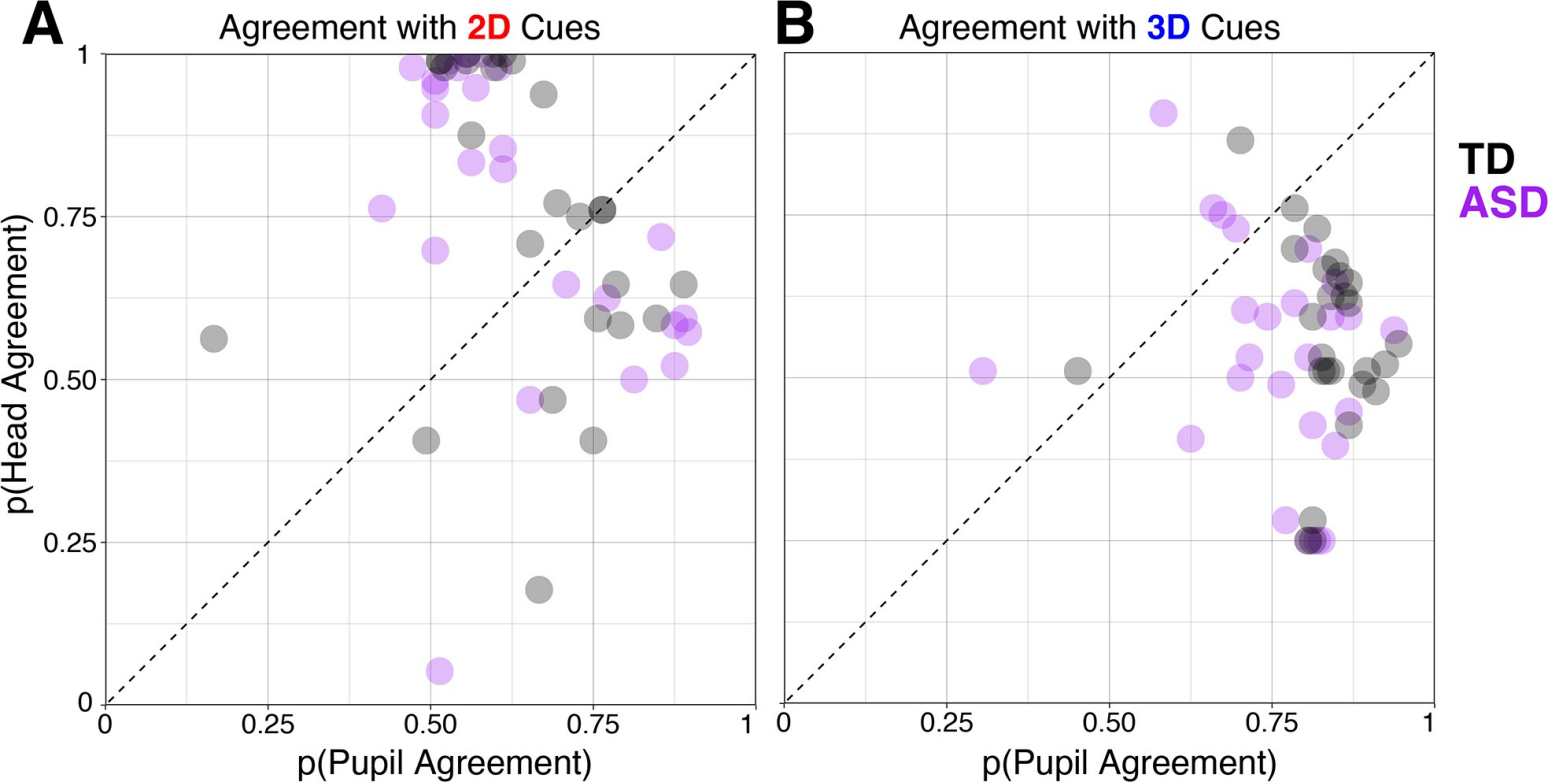
Agreement of observers’ responses with pupil and head cues in the two-dimensional condition (a) and three-dimensional condition (b). Typically-developing observers are illustrated with gray while observers with ASD are illustrated with purple. Axes reflect the probability that the observer’s judgments corresponded with the information provided by that cue across all trials.

Next, we analyzed how often observers’ judgments of gaze direction agreed with the pupil or head cues while accounting for ASD status (Fig 4). We were interested in categorical judgements—how often each observer said “left” when the pupils were pointing to the left, and “right” when pupils were pointing to the right (or when the head was to the left etc.). For each observer, we calculated the proportion of judgments that “agreed” with the pupil cues (disregarding the head), or that “agreed” with the head cues (disregarding the pupils). We did this separately for trials from the 2D and 3D conditions, and in this case, we took into account whether each observer received a diagnosis of ASD. This analysis also allowed us to get a sense for the patterns of cue usage in the 2D and 3D conditions.

As visual inspection of the distribution of the percent agreement values suggested potential non-normality due to clustering of points near the upper bound possible value of 1, we used conservative non-parametric tests for analysis. In the 2D task, the degree to which observers’ judgments agreed with pupil cues was significantly greater than chance (M = 0.64; non-parametric Wilcoxon signed rank test versus p = 0.5, V = 1249.5, p = 4 × 10^−8^), as was mean agreement between judgments and informative head cues (M = 0.75, non-parametric Wilcoxon signed rank test, V = 1190.5, p = 9 × 10^−8^). A negative correlation between agreement with pupil cues and with head cues (non-parametric Spearman’s Rho, r(49) = -0.46, p = 0.0007) indicated that observers’ individual judgments may have traded off between cue information, though on average, judgments accorded more with head cues than with pupil cues (Wilcoxon paired signed rank test, V = 380, p = 0.008). In the 2D task, there were no significant differences between agreement with pupil or head cues among observers with and without ASD (pupil cue agreement: non-parametric Wilcoxon Rank Sum test *p* = 0.46; head cue agreement: non-parametric Wilcoxon Rank Sum test *p* = 0.89).

In contrast to the negative correlation between judgment agreement with pupil cues versus head cues in the 2D task, agreement of judgments with cues was uncorrelated in the 3D task (Spearman’s Rho, r(49) = -0.14, p = 0.32). This can be, at least partially, explained by noting that while agreement with pupil cues was quite high in the 3D task (M = 0.79; Wilcoxon signed rank test versus 0.5, V = 1318.5, p = 8 × 10^−10^), agreement with head cues was relatively low (M = 0.55; Wilcoxon signed rank test versus 0.5, V = 863.5, p = 0.03). Note that not using a cue at all would lead to an average agreement of 0.5, indicating that head rotation information in the 3D condition had a weak influence at best, in contrast with a significantly greater reliance on pupil cues (Wilcoxon paired signed rank test, V = 1248, p = 4 × 10^−8^), a reversal of the pattern from the 2D task. Agreement with pupil cues was significantly lower in the 3D task for observers with ASD than typically-developing observers (two-sample t-test, *t*(49) = 2.45, *p* = 0.018; Wilcoxon Rank Sum test, W = 167, *p* = 0.003; M_ASD_ = 0.75, M_TD_ = 0.83; Cohen’s d = 0.68), but there was no significant difference between head agreement among observers with or without ASD in the 3D task (non-parametric Wilcoxon Rank Sum test *p* = 0.53). Clearly, both TD and ASD observers used the eyes reliably when evaluating a 3D face, but those with ASD tended to do so less effectively.

Comparisons across the 2D and 3D conditions are potentially complicated by differences in the two tasks (see the Supplemental Materials for tests of agreement between judgments and cues across conditions, e.g., agreement of gaze judgments with pupil cues in 2D vs. 3D).

### Logistic regression: Model series 1

We next analyzed our data using a multi-level logistic model [42]. This allowed us to quantify individual differences and examine the effect of head and pupil cues across our groups, while pooling our data for maximum statistical power. We performed the regression in R (version 3.4.1) using the lme4 package (version 1.1–19). We fit two series of models. Model Series 1 predicted individuals’ “rightward” judgments with fixed effects for intercept, head cues, and pupil cues, and random effects (i.e., individual differences from the group-level fixed-effects) for intercept, head cues, and pupil cues (see Supplementary Materials for details of model-fitting). We ran separate model fits in Model Series 1 for data from the 2D and 3D conditions. We included no assumptions about group membership (TD vs ASD) in Model Series 1. The regression weights (or β values) in this model indicate the extent to which head or pupils influence binary leftward/rightward judgments of gaze direction.

**Fig 5:**
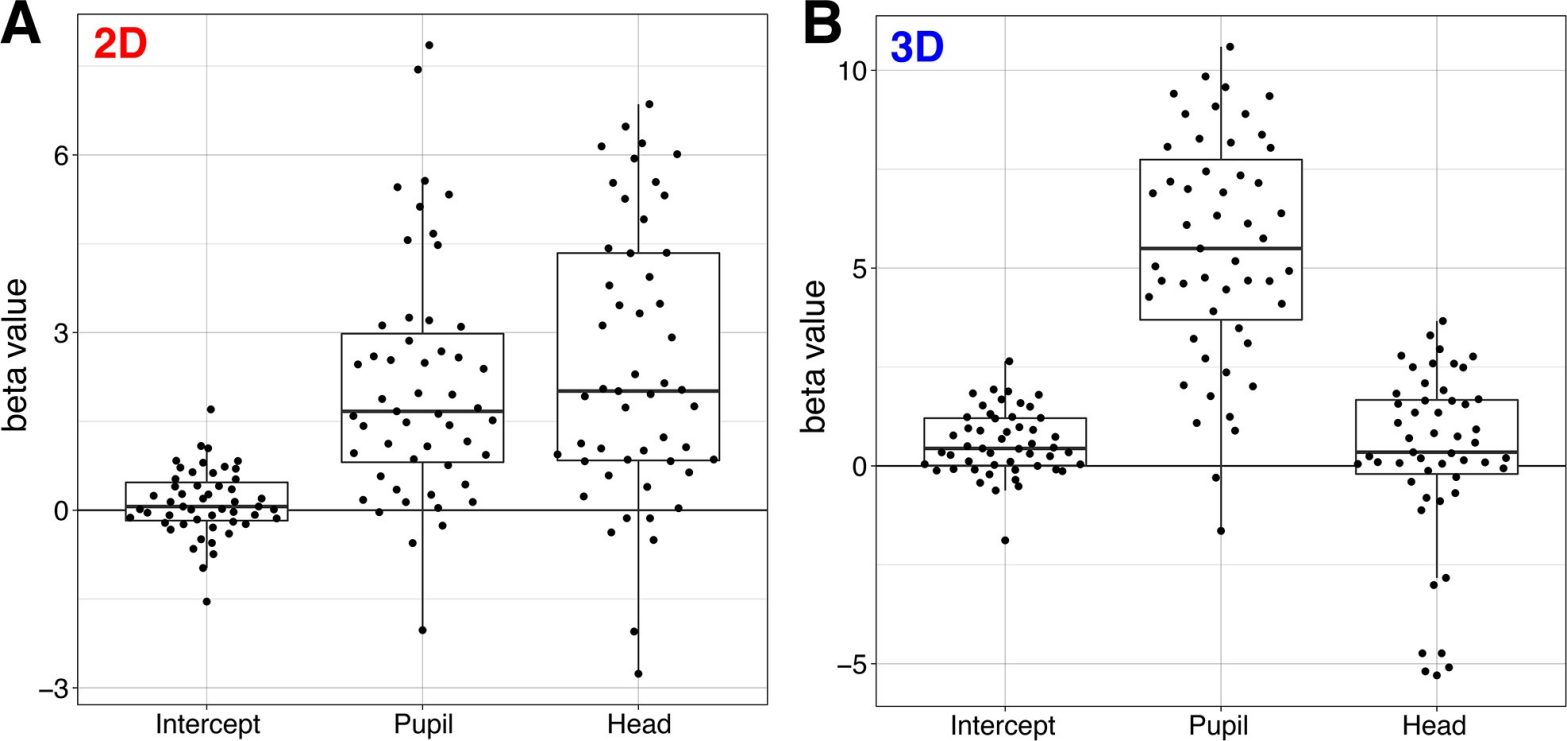
Boxplots of the estimated parameters. A) Estimates from the 2D condition indicate very strong and consistent effects of pupil shifts and head rotations, but not intercept. B) Estimates from the 3D condition indicate very strong and consistent effects of pupil direction, but not head direction, and a small positive effect of intercept.

Fitting Model Series 1 to data from the 2D condition revealed significant effects for head rotation (β = 2.62, SE = 0.37, *p* < .001), and pupil shifts (β = 2.22, SE = 0.32, *p* < .001), but not the intercept (*p* = 0.18). Fig 5A shows each observer’s β values for the effects of intercept, head rotations, and pupil shifts on gaze perception. This figure illustrates strong overall effects of head and pupil use for judgments of 2D faces, but also considerable individual differences in the way observers integrated head and pupil cues in our task. The lack of an effect of intercept indicates that observers had no overall bias for reporting gaze as rightward or leftward.

Fitting Model Series 1 to data from the 3D condition revealed a different pattern of results. We found significant effects for intercept (β = 0.61, SE = 0.14, *p* < .001) and pupil direction (β = 5.83, SE = 0.49, *p* < .001), but not head direction (*p* = 0.45). Fig 5B illustrates the strong overall effect of pupil use for judgments of 3D faces in our task, and again, considerable individual differences in the way observers integrated head and pupil directions. The positive effect of intercept indicates that observers had an overall bias for reporting gaze as rightward in this case. In terms of the size of this bias, participants would on average say “right” 64% of the time on a hypothetical trial on which the pupil and the head were completely uninformative (i.e., straight ahead). The effect of head direction was not significant at the group level for judgments of 3D gaze. However, we noted the considerable variability in β values for head-use, including some very negative scores. Clearly, some individual observers’ evaluations of 3D gaze were influenced by changes in head rotation.

### Logistic regression: Model series 2

To explore these individual differences further, including ASD status, we fit fully independent individual-level models to each observer’s data. This analysis allowed us to evaluate the significance of parameter estimates for each observer, and to get a sense for how this depended on ASD status. Rather than focusing on the magnitude of parameter estimates, we simply counted the number of observers showing significant effects of head rotation, pupil rotation, and intercept, separately for the 2D and 3D data, according to ASD status. The results of this analysis are shown in Table 1 for 2D data and Table 2 for 3D data. The effects of intercept, head, and pupil rotations were generally split equally between the two groups. These tables also highlight the individual variability in the extent to which changes in head and pupil rotations influenced judgments of gaze direction. Lastly, Table 2 shows that although there was no effect of head rotation at a group level, many observers’ judgments of gaze were influenced by changes in head rotation, with some observers even showing negative effects of head rotation.

**Table 1:** Number of individuals with significant effects of intercept, head, and pupils in 2D.

2D	Intercept			Head			Pupil		
	*Total*	*ASD*	*TD*	*Total*	*ASD*	*TD*	*Total*	*ASD*	*TD*
**Sig. positive**	13	5	8	36	18	18	37	16	21
**n.s.**	31	15	16	12	6	6	12	8	4
**Sig. negative**	7	5	2	3	1	2	2	1	1

**Table 2:** Number of individuals with significant effects of intercept, head, and pupils in 3D.

3D	Intercept			Head			Pupil		
	*Total*	*ASD*	*TD*	*Total*	*ASD*	*TD*	*Total*	*ASD*	*TD*
**Sig. positive**	24	11	13	24	11	13	44	21	23
**n.s.**	25	12	13	20	10	10	6	3	3
**Sig. negative**	2	2	0	7	4	3	1	1	0

### Exploration of determinants of individual differences in performance

The analysis above illustrates considerable variability in gaze perception across individuals. Factors such as age, visuospatial reasoning, and verbal reasoning may account for some of this variability, especially in combination with ASD status. Although we were not powered to fully examine and dissociate all these factors in combination with ASD status, we ran exploratory models to get a sense for how they might interact. Briefly, in both the 2D and 3D tasks, simple regressions featuring age outperformed models that instead included scores of visuospatial or verbal abilities. Thus, the way in which changes in these cues influenced observers’ judgements of others’ gaze direction was not particularly strongly associated with cognitive skills in our study. Of note, cognitive scores were broadly average in this study for both typically-developing observers (WISC-V VCI SS = 117, SD = 15; VSI SS = 118, SD = 13) and observers with ASD (WISC-V VCI SS = 107, SD = 18; VSI SS = 104, SD = 18). The results of the ‘full’ models, incorporating all covariates, were similar; the influence of changes in pupil rotation on gaze judgments increased with age across 2D and 3D tasks, even when accounting for ASD status as well as visuospatial and verbal abilities, but the influence of changes in head rotation on gaze judgments did not follow this same pattern with age. These findings should be interpreted with caution; please refer to the Supplementary Materials for detailed results.

## Discussion

We showed that children and adolescents with ASD and their typically-developing peers can perceive gaze as an emergent visual feature. When looking at 2D faces, changes in both head and eye rotations influenced their judgments of gaze direction, although there were considerable individual differences in cue prioritization. When looking at 3D faces, changes in eye rotations influenced judgments of gaze more strongly than changes in head rotations, although still with notable individual differences. Lastly, all observers relied more heavily on changes in eye rotations than head rotations to evaluate gaze on 3D faces, but this difference was attenuated in individuals with ASD.

Gaze perception is a complex and underspecified process. This is especially so for people with ASD [43], who produce different patterns of intact or atypical performance across several domains [44]. Even less is known about gaze perception in children and adolescents with ASD. Our findings thus address an overlooked question about whether individuals with ASD perceive gaze as an emergent feature. They do, albeit with some individual variability. This is especially important when considering that individuals with ASD have difficulties with global or holistic perception [45, 46].

Exploratory analyses indicated that regardless of ASD status, older observers’ judgments of gaze direction were more strongly influenced by changes in eye rotation, consistent with our previous work with the same 2D faces, and highlighting the value of looking at gaze processing through a developmental lens [7]. In addition to chronological age, it is also important to consider cognitive level. There is a wide spectrum of cognitive and adaptive functioning across individuals with ASD, which may account for some of the variability in findings across the ASD literature. For example, observers with ASD who showed difficulties with shifting attention in response to a head turn, also had “lower mental age” relative to their peers without ASD [29]. Individual differences in communication skills may also in part explain mouth fixations in some ASD samples [25]. A strength of our study is that we carefully assessed ASD symptomology and cognitive abilities for both the typically-developing and atypically-developing observers in our sample. When we accounted for individual differences in these factors, our findings suggest that age, more than verbal or visual skills, impacts the amount of influence from changes in pupil and head rotations on an observer’s judgments of gaze direction, at least among those with broadly average cognitive skills. This is consistent with other studies showing that children with ASD (but without cognitive impairments) may process eye gaze similarly to their typically-developing peers [28].

Our analytical approach allowed us to quantify how much variability in any individual observer’s judgments of gaze direction were influenced by *changes* in a face’s head or pupil rotation. Our results therefore speak to the influence of these features across many trials and perceptual moments, and they support inferences about whether changes in one cue were more impactful on an individual’s perception than another cue, overall. For example, it would be fair to conclude that a given observer’s variability in gaze judgments was more strongly influenced by changes in pupil rotation than head rotation. One should not, however, conclude that such an observer did not use head rotations to make their judgments. Face and gaze perception is inherently holistic whereby head rotations influence the aperture of the eyes [13–15], and all judgments of gaze in our task were made in the context of heads. We thus stress that observers still used head information in every judgment in our task, no matter how much their judgments across many trials might have been influenced by changes in pupil rotation.

Our analytical approach also allowed us to capture influence from changes in head and pupil rotations on gaze judgments on top of any leftward or rightward biases individual observers might have had. Although we did not predict systematic biases at the group level, we did find a bias to report “rightward” gazes in the 3D task. This was not due to any technical issue with the precision of the robot’s gaze directions (otherwise all observers should have produced a rightward bias), or placement of the robot (the experimenter carefully lined up the robot in front of the observer’s seat at the start of each session). Rather, we believe that some observers might have leaned to their left during the experiment despite the experimenter’s instructions to remain centered and to hold still. Allowing observers some freedom of movement was a compromise we had to make since restraint in a chin rest, for example, would have been especially difficult for children sensitive to tactile stimulation (common among individuals with ASD). Nevertheless, our results focus on how changes in gaze perception result from changes in head and pupil rotations, and these effects occur independently of bias.

Throughout out analyses, we reported on results from the 2D and 3D tasks separately. For example, we found that within the 2D task, observers whose judgments were most strongly influenced by pupil rotations were less influenced by changes in head rotations. It appears that observers may have engaged in a sort of a tradeoff, prioritizing changes in one cue or the other to drive their judgments of gaze direction. We analyzed data from the 2D and 3D tasks separately as part of a conservative analytic approach because there were notable differences between these conditions beyond the addition of stereoscopic depth cues that made direct comparison between these datasets interpretationally challenging. First, we controlled the eye apertures of the faces in the 2D condition so that they did not change as a head rotated. This allowed us to capture a pure attractive effect from the rotation of the head on perceived gaze direction (a visual illusion known as the Wollaston effect), which is often evaluated in examinations of 2D gaze [8, 47]. In contrast, the shapes of the eye apertures were necessarily uncontrolled in the case of the 3D faces. That is, changes in head rotation in the 3D task changed both the shape of the head and the eye apertures, which have been shown to have attractive and repulsive influences on perceived gaze direction, respectively [15, 34]. Therefore, the origins of the effects we isolated in the 2D- and 3D- conditions were not completely identical. Second, we used a schematized image of a face in the 2D condition rather than a 2D photograph of the robot. We took this approach for the sake of continuity with a recent report from our team that included a developmental sample and an unvalidated exploratory sample of children with ASD [7]. Third, the robot’s head subtended an image on the observer’s retinae roughly twice as large as the image of the 2D head. And fourth, observers were more squarely situated in the role of spectator in the 2D condition than in the 3D condition, where one could argue they were involved in a pseudo-interaction with the robot. This could have had consequences in terms of activating processes unique to social cognition over and above more straightforward visual perceptual processes [37]. We thus consider the 2D and 3D tasks as two mostly distinct examinations of gaze perception. Nevertheless, our within-subjects design affords the possibility of direct quantitative examination of relationships across the 2D and 3D tasks. Briefly, we found that the influence of head cues was strongly correlated across 2D and 3D tasks, while pupil cue influence was only marginally related. Additionally, the influence of head rotations was smaller in the 3D task compared with the 2D task. This may have been due, at least in part, to a repulsive influence on perceived gaze direction from the changing apertures of the eyes in the 3D condition [33] diminishing an attractive effect from the head, an effect that could not have occurred in the 2D task in which the eye apertures did not change. For detailed results of these exploratory analyses and discussion thereof, see the Supplementary Materials.

We found that perceived gaze was consistently attracted to the rotations of the pupils and heads in the 2D task, which is consistent with previous work [8, 47]. Yet unlike previous examinations of 3D gaze which demonstrated a repulsive effect from head rotations of real “lookers” seated in front of observers [48–50], most of the observers in our 3D condition still experienced a subtle attractive effect from rotations of the robot’s head (Fig 3D). It is unclear why our results differ from these other reports. Some potentially relevant factors include viewing distance (50cm in our design vs. 84-500cm in these earlier investigations), amount of head rotation (8° in our design vs 10–30°), or even the binary (left vs. right) nature of our response option. It may be the case that head and eye interactions in the perception of gaze may be less consistent than previously thought, or subject to boundary conditions that could be mapped out in more detail by additional investigation.

There are, of course, outstanding questions beyond the scope of this paper and additional limitations of our experimental design, which should be considered before generalizing our findings. First, although we took into account both age and cognitive skills, our findings may not apply to pre-school-aged children, girls with ASD, or children with ASD with co-occurring conditions (e.g., language delays, intellectual disability). It will thus be important for future research to examine emergent gaze in these populations. Second, although observers in our study perceived gaze emergently, many individuals with ASD nonetheless have difficulties with gaze. It is thus important to consider differences between the demands of tasks in this study and other, more complex social interactions. We did not evaluate whether observers spontaneously followed gaze cues or fixated their own gaze. Since our study does not focus on direct gaze, it does not shed light on whether individuals with ASD appropriately modulate eye contact. Of note, one of the proposed explanations for difficulties with eye contact in ASD is that social interactions may evoke anxiety and elicit gaze avoidance [23], which we purposefully minimized here through interactions with a robot. Conversely, individuals with ASD in our sample may have been more interested in the robot than their counterparts without ASD. Without subjective reports of how observers felt about the robot or objective measures of their attention (e.g., fixation patterns from eye-tracking), we can only speculate about how these sorts of unintended consequences might have played out in our results. In any case, observers in our task were freed from the burden of theorizing about the robot’s attention, nor was there potential or expectation for observers to initiate joint attention since the robot could not react to their eye movements. Had we instead used a real human, it would have been unclear if any differences were due to the 3D context or the more complex social demands of the task. Replicating our task with a real 3D human would be of great interest, but for this investigation, we opted to incrementally “scale up” from the relative simplicity of more common computer-based tasks, minimizing the added difficulty of conducting 2^nd^-person interactive research [35], or real-life “everyday attention” [36]. In fact, our own recent work illustrates the increases in sensitivity to gaze cues from the head and eyes that can be expected when scaling up from a virtual avatar to a virtual robot, a physically present robot, and finally, a physically present human [51].

Our findings show the importance of evaluating gaze as an integrated feature that accounts for information beyond the eyes. Evaluating gaze perception in 2D and 3D faces also paves the way for understanding human-robot assisted interactions, which are being increasingly developed for healthcare and commercial purposes. Lastly, we proposed an approach that considers individual variability in addition to group differences. Gaze perception is a complex and flexible process, and it is important to study it through a developmental lens in clinical and typically-developing populations.

## Supporting information

S1 File (DOCX)
