# Supplementary material for: Gaze perception from head and pupil rotations in 2D and 3D: Typical development and the impact of autism spectrum disorder
Source: PLoS One. 2022 Oct 27;17(10):e0275281. doi: 10.1371/journal.pone.0275281 (PMC9612464; doi:10.1371/journal.pone.0275281)
Supplement: S1 File — (DOCX) [file pone.0275281.s001.docx]

**Supplemental Online Material**

**Method**

# Assessment. Caregivers also completed the Adaptive Behavior Assessment System, Third Edition (ABAS-3), a measure of adaptive functioning across development (1) (as well as two screeners for ASD, the Social Responsiveness Scale, Second Edition (2), and the Social Communication Questionnaire (SCQ) (3). We did not analyze data from these measures as we prioritized other variables in our logistic regression models that were more central to our research questions.

**Table S1*.*** Summary of participant demographics and assessments (with mean, standard deviation, and range values where appropriate, and the number of participants contributing).

|  | **TD (N = 26)** | | | | **ASD (N = 25)** | | | |
| --- | --- | --- | --- | --- | --- | --- | --- | --- |
|  | *Mean* | *S.D.* | *Range* | *N* | *Mean* | *S.D.* | *Range* | *N* |
| **Age** (years) | 10.5 | 2.2 | 8 - 15 | 26 | 10.7 | 2.6 | 7 - 16 | 25 |
| **Sex** | 9 male | 17 female |  | 26 | 6 male | 19 female |  | 25 |
| **VCI** | 117.0 | 15.1 | 81 - 146 | 24 | 107.1 | 18.3 | 68 - 139 | 22 |
| **VSI** | 118.4 | 12.8 | 94 - 138 | 24 | 104.3 | 18.4 | 78 - 141 | 22 |
| **FSIQ** | 116.1 | 14.3 | 78 - 140 | 24 | 100.5 | 17.4 | 74 - 129 | 22 |
| **ABAS** | 96.1 | 9.9 | 73 - 119 | 24 | 75.7 | 10.5 | 48 - 101 | 23 |
| **SCQ** | 2.5 | 2.1 | 0 - 8 | 24 | 17.9 | 6.3 | 7 - 31 | 24 |
| **ADOS** | 1.0 | 0.4 | 0 - 2 | 22 | 6.8 | 2.0 | 4 - 10 | 19 |

VCI = Verbal Comprehension Index; VSI = Visual Spatial Index; FSIQ = Full Scale Intelligence Quotient; ABAS = Adaptive Behavior Assessment System, General Adaptive Composite; SCQ = Social Communication Questionnaire; ADOS = Autism Diagnostic Observation Schedule, Comparison Score.


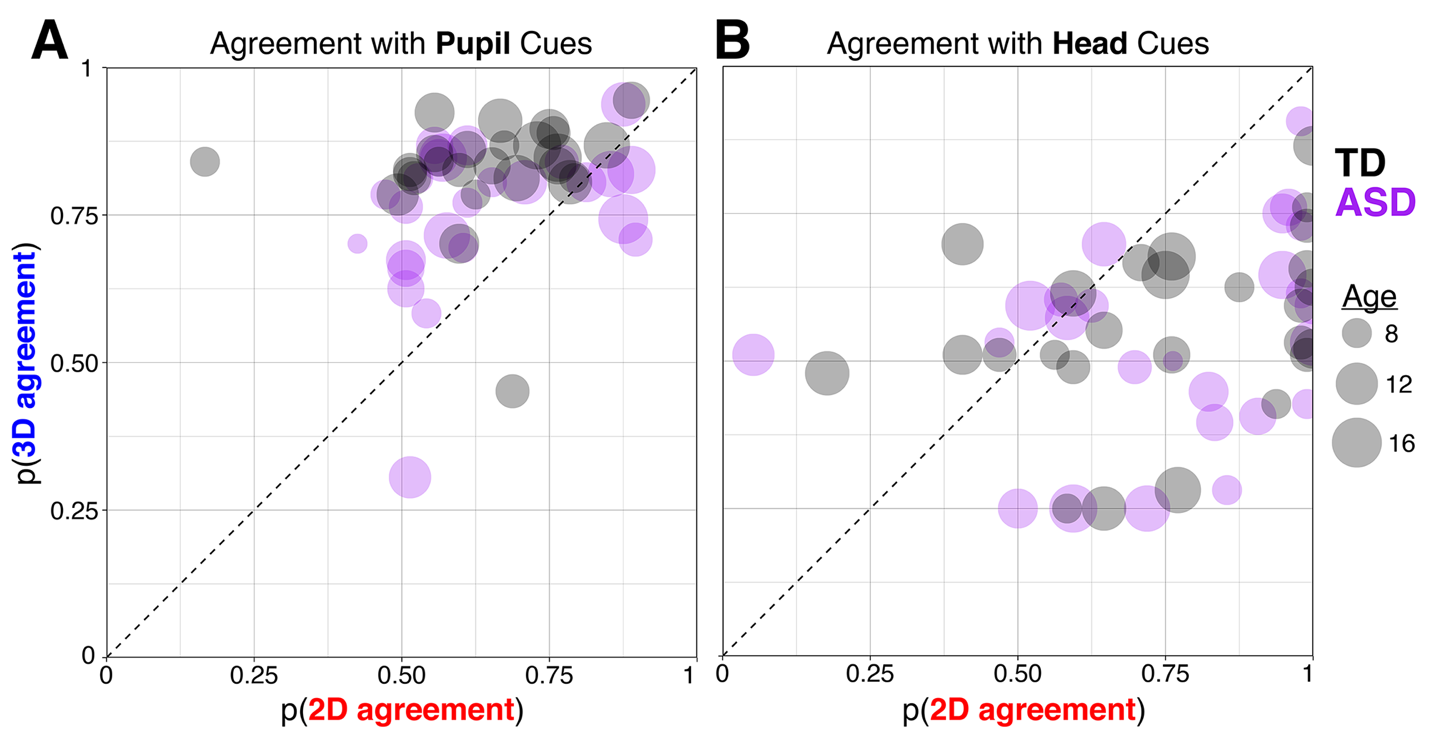


**S1 Fig. Agreement of observers’ responses with pupil cues (a), and head cues (b).** Typically-developing (TD) observers are illustrated with gray while observers with ASD are illustrated with purple. The size of each dot corresponds to each observer’s age. Axes reflect the probability that the observer’s judgments corresponded with the information provided by that cue across all trials.

# Logistic Regression Models

**Diagnosing ASD.** We treated ASD as a binary variable (1 if the individual had been diagnosed with Autism Spectrum Disorder and performed above the cut-off score on ADOS-2, 0 if typically-developing). We linearly rescaled Verbal Comprehension Index (VCI) and Visual Spatial Index (VSI) scores to be on a 0-1 scale. Individuals missing VCI or VSI scores had their scores replaced with a ‘0’, so that VCI/VSI estimates did not affect these observers’ scores. A total of 5 observers’ scores were replaced with ‘0’ either because (a) their families were unable to attend the session, (b) they provided a WISC-IV report (which provides a perceptual reasoning index (PRI) rather than VSI and FRI scores), and (c) one observer was unable to complete all of the subtests due to emotion and behavior regulation difficulties, which resulted in the session being discontinued.

**Exploratory Model with ASD, VCI, VSI, and Age.** We ran an exploratory model using data from the 2D and 3D tasks that incorporated potential covariates of ASD, including age, visuospatial skills (WISC-V VSI score), and verbal skills (WISC-V VCI score). We chose to examine VCI and VSI variables instead of the full scale IQ (FSIQ) for a number of reasons. First, it would not be appropriate to report FSIQ for many of our observers whose index scores were significantly different across domains, yielding inaccurate FSIQ scores. Second, we were more interested in core reasoning skills than working memory (WMI) and processing speed (PSI), which are typically conceptualized as efficiency skills that support but do not reflect “intelligence.” Lastly, we chose verbal (VCI) and visuospatial (VSI) indices (over the fluid reasoning index [FRI]) as these skills were the most relevant to the perception of emergent gaze—a visual illusion with a social-communicative purpose.

## Results

**Comparing Performance in the 2D and 3D tasks.** Care must be taken when comparing the two- and three-dimensional conditions in the current study as there were several differences across these conditions that may affect interpretation (for example, the aperture of the eyes within the face was controlled in the 2D task to be invariant with respect to head rotation, while it was not in the 3D task). With this caveat, however, one can compare the influence of these cues across conditions to get a sense of how these cues may differentially affect judgments in 2D and 3D contexts.

Pupil agreement was correlated across 2D and 3D tasks (Spearman’s non-parametric rho = 0.34, *p* = 0.02), while observers’ responses agreed more with pupil cues on average in the 3D task compared to the 2D task (paired t-test *t*(50) = 6.6, *p* < .001; M_2D_ = 0.64; M_3D_ = 0.79; Cohen’s d = 0.93).

For TD individuals, agreement of judgments with pupil cues was uncorrelated between the 2D and 3D conditions (r(24) = 0.11, p = 0.61), and was significantly greater for the 3D vs. 2D case (t(25) = 5.67, p = 6.6e-6). For ASD individuals, the influence of pupil rotations was marginally correlated across conditions (r(23) = 0.38, p = 0.06), while their judgments agreed with pupil cues more in the 3D condition versus the 2D condition (t(24) = 3.73, p = 0.001) as with TD individuals. Together these findings indicate that judgments more closely aligned with pupil cues in the 2D than the 3D condition, and there may be some weak relationship between the influence of pupils on judgments in the two conditions.

As many participants experienced near-ceiling levels of agreement of their rightward judgments with head cue direction in the 2D condition, to compare the influence of these cues across 2D and 3D cases, we use non-parametric statistics out of an abundance of caution. Collapsing across the TD and ASD groups, agreement of participants’ rightward judgments with head information was significantly correlated (Spearman’s rho; r(49) = 0.34, p = 0.016; note that the Pearson’s correlation identified a nearly identical relationship) and was significantly greater in the 2D versus 3D condition (Wilcoxon signed rank test, p = 1.7e-6; a paired t-test identified a nearly identical result).

Repeating the analyses separately within TD and ASD individuals identified similar patterns. TD individuals’ head cue agreement was correlated across 2D and 3D conditions (Spearman’s Rho; r(24) = 0.41, p = 0.04), and significantly greater in the 2D versus 3D conditions (Wilcoxon signed rank test, p = 0.0007), while ASD individuals’ head cue agreement was similarly related, but not at a significant level across conditions (Spearman’s Rho; r(24) = 0.31, p = 0.13). Like TD individuals, ASD individuals’ judgments agreed with head cues more in the 2D versus 3D condition (Wilcoxon signed rank test, p = 0.0008). Thus, agreement of judgments with head cues appeared somewhat related across domains, and significantly greater in the 2D condition versus the 3D condition.

To summarize, head usage was strongly related, while pupil usage was only marginally related, across the 2D and 3D modalities. And in the 3D task, observers’ judgments were more strongly influenced by changes in pupil rotations compared with changes in head rotations. This may have occurred because the eyes on the 3D robot were larger than the eyes on the 2D face, and overall, the robot’s face in the 3D condition produced a large image on an observer’s retinae than the 2D faces in the computer task. This is relevant because people rely more on the eyes than the head to evaluate gaze in close face-to-face interactions (4,5). Alternatively, the 3D robot was more interactive than the 2D face, which may have increased observer engagement and pupil usage (6). Nevertheless, these differences across the 2D and 3D conditions are still interesting because they suggest that gaze perception is a flexible process—not only are there individual differences in the extent to which people rely on head versus pupil rotations, but individuals may also prioritize different cues under different conditions. The importance of seeing gaze across 2D and 3D contexts does not waiver, but the cues people use in these contexts can change, and they seem to vary considerably across individuals.

## Models of Choices in the Two-Dimensional (2D) Task. The following models incorporated predictors for usage of head rotation information, pupil rotation information, and separate interactions of those terms with ASD and with covariates, including Age, VCI score, and VSI score. These models were exclusively of judgments in the 2D task. Below, we include the R commands used to run the analyses, statistical outputs of the models, and summaries of the results.

**Basic 2D Model: No Covariates (*model from main text*)**

glmer(rightward ~ 1 + headrs + pupilrs + (1 + headrs + pupilrs | subnum), data = d[d$robot==0,], family='binomial', control = glmerControl(optimizer="bobyqa", optCtrl=list(maxfun=100000)));

**AIC:** 5543.1

**Fixed effects:**

Estimate Std. Error z value Pr(>|z|)

(Intercept) 0.12969 0.09579 1.354 0.176

**headrs 2.62013 0.37286 7.027 2.11e-12 *****

**pupilrs 2.22211 0.31770 6.994 2.66e-12 *****

**Model E1: Incorporating ASD Status and Age**

glmer(rightward ~ 1 + headrs*age + headrs*asd + pupilrs*age + pupilrs*asd + (1 + headrs + pupilrs | subnum), data = d[d$robot==0,], family='binomial', control = glmerControl(optimizer="bobyqa", optCtrl=list(maxfun=100000)));

**AIC:** 5536.9

**Fixed effects:**

Estimate Std. Error z value Pr(>|z|)

(Intercept) -0.16046 0.42227 -0.380 0.703949

**headrs 4.93772 1.64723 2.998 0.002721 ****

age 0.04236 0.03826 1.107 0.268293

asd -0.32565 0.18361 -1.774 0.076137 .

pupilrs -1.92058 1.27339 -1.508 0.131492

headrs:age -0.19843 0.14872 -1.334 0.182144

headrs:asd -0.47202 0.72038 -0.655 0.512318

**age:pupilrs 0.40448 0.11601 3.487 0.000489 *****

asd:pupilrs -0.33047 0.54871 -0.602 0.546996

**Model E2: Incorporating ASD Status and VSI**

glmer(rightward ~ 1 + headrs*asd + headrs*VSIrsNoNA01 + pupilrs*asd + pupilrs*VSIrsNoNA01 + (1 + headrs + pupilrs | subnum), data = d[d$robot==0,], family='binomial', control = glmerControl(optimizer="bobyqa", optCtrl=list(maxfun=100000)));

**AIC:** 5550.7

**Fixed Effects:**

Estimate Std. Error z value Pr(>|z|)

**(Intercept) 0.30679 0.13623 2.252 0.0243 ***

**headrs 2.91525 0.54713 5.328 9.91e-08 *****

asd -0.36268 0.20176 -1.798 0.0722 .

VSIrsNoNA01 -0.10564 0.21055 -0.502 0.6159

**pupilrs 2.26676 0.45735 4.956 7.18e-07 *****

headrs:asd -0.59338 0.80584 -0.736 0.4615

headrs:VSIrsNoNA01 -0.19889 0.83961 -0.237 0.8127

asd:pupilrs -0.09421 0.67768 -0.139 0.8894

VSIrsNoNA01:pupilrs 0.50471 0.71028 0.711 0.4773

**Model E3: Incorporating ASD Status and VCI**

glmer(rightward ~ 1 + headrs*asd + headrs*VCIrsNoNA01 + pupilrs*asd + pupilrs*VCIrsNoNA01 + (1 + headrs + pupilrs | subnum), data = d[d$robot==0,], family='binomial', control = glmerControl(optimizer="bobyqa", optCtrl=list(maxfun=100000)));

**AIC:** 5548.9

**Fixed Effects:**

Estimate Std. Error z value Pr(>|z|)

**(Intercept) 0.32597 0.13132 2.482 0.0131 ***

**headrs 2.78911 0.53070 5.256 1.48e-07 *****

**asd -0.40366 0.19108 -2.112 0.0346 ***

VCIrsNoNA01 -0.37951 0.26062 -1.456 0.1453

**pupilrs 2.35278 0.44811 5.250 1.52e-07 *****

headrs:asd -0.33213 0.76888 -0.432 0.6658

headrs:VCIrsNoNA01 0.85472 1.04089 0.821 0.4116

asd:pupilrs -0.27574 0.64883 -0.425 0.6709

VCIrsNoNA01:pupilrs 0.03213 0.88788 0.036 0.9711

**Model E4: Incorporating ASD Status, Age, VSI, and VCI**

glmer(rightward ~ 1 + headrs*age + headrs*VSIrsNoNA01 + headrs*asd + headrs*VCIrsNoNA01 + pupilrs*age + pupilrs*VSIrsNoNA01 + pupilrs*asd + pupilrs*VCIrsNoNA01 + (1 + headrs + pupilrs | subnum), data = d[d$robot==0,], family='binomial', control = glmerControl(optimizer="bobyqa", optCtrl=list(maxfun=100000)));

**AIC:** 5544.8

**Fixed Effects:**

Estimate Std. Error z value Pr(>|z|)

(Intercept) -0.13052 0.41673 -0.313 0.754130

**headrs 4.97164 1.63326 3.044 0.002335 ****

age 0.04344 0.03759 1.156 0.247815

VSIrsNoNA01 -0.01364 0.21412 -0.064 0.949200

**asd -0.41321 0.19937 -2.073 0.038211 ***

VCIrsNoNA01 -0.38248 0.26908 -1.421 0.155192

pupilrs -1.98765 1.25929 -1.578 0.114476

headrs:age -0.20332 0.14683 -1.385 0.166137

headrs:VSIrsNoNA01 -0.45646 0.84967 -0.537 0.591115

headrs:asd -0.42731 0.79102 -0.540 0.589060

headrs:VCIrsNoNA01 1.02792 1.05822 0.971 0.331366

**age:pupilrs 0.40315 0.11431 3.527 0.000421 *****

VSIrsNoNA01:pupilrs 0.54273 0.64778 0.838 0.402128

asd:pupilrs -0.17123 0.59689 -0.287 0.774210

VCIrsNoNA01:pupilrs -0.23249 0.80781 -0.288 0.773497

**Summary of 2D Results with Covariates.** Of the simple (only one continuous covariate) models, Models E1, E2, and E3, Model E1 with ASD and age performed best, compared to models that featured VCI or VSI in place of age. In terms of cue usage, head usage was a strong factor across all models, while pupil usage appeared to be a similarly strong factor alone (or in interaction with Age if Age was present). ASD only appeared to have an effect on the overall bias away from making “rightward” judgments, and so did not appear to consistently affect the usage of head or pupil cues when determining gaze direction. In the ‘full’ model, Model E4, incorporating all covariates (ASD, age, VSI score, and VCI score), head usage was consistently present, while pupil usage increased with age. ASD’s only effect was for a ‘leftward’ bias, interacting neither with head cue usage or pupil cue usage. VSI & VCI also had no main effects or interactions. Note that Model E1 outperformed all other models on the basis of AIC (i.e. its AIC value was lowest, indicating the ‘best’ tradeoff between model complexity and model fit).

## Models of Choices in the Three-Dimensional (3D) Task. The following models incorporated predictors for usage of head rotation information, pupil rotation information, and separate interactions of those terms with ASD and with covariates, including Age, VCI score, and VSI score. See above for variable information. These models were exclusively of judgments in the 3D task. Below, we include the R commands used to run the analyses, statistical outputs of the models, and a summaries of the results.

**Basic 3D Model: No Covariates (*model from main text*)**

glmer(rightward ~ 1 + headrs + pupilrs + (1 + headrs + pupilrs | subnum), data = d[d$robot==1,], family='binomial', control = glmerControl(optimizer="bobyqa", optCtrl=list(maxfun=100000)));

**AIC:** 4578.9

**Fixed effects:**

Estimate Std. Error z value Pr(>|z|)

**(Intercept) 0.6051 0.1366 4.429 9.47e-06 *****

headrs 0.2623 0.3464 0.757 0.449

**pupilrs 5.8317 0.4874 11.964 < 2e-16 *****

**Model E5: Incorporating ASD Status and Age**

glmer(rightward ~ 1 + headrs*age + headrs*asd + pupilrs*age + pupilrs*asd + (1 + headrs + pupilrs | subnum), data = d[d$robot==1,], family='binomial', control = glmerControl(optimizer="bobyqa", optCtrl=list(maxfun=100000)));

**AIC:** 4572.7

**Fixed Effects:**

Estimate Std. Error z value Pr(>|z|)

(Intercept) 0.90983 0.60796 1.497 0.13452

headrs 1.40603 1.55231 0.906 0.36506

age -0.01359 0.05540 -0.245 0.80621

asd -0.31584 0.26343 -1.199 0.23054

pupilrs 1.28562 1.95409 0.658 0.51059

headrs:age -0.07125 0.14139 -0.504 0.61433

headrs:asd -0.78879 0.68062 -1.159 0.24649

**age:pupilrs 0.52899 0.18072 2.927 0.00342 ****

**asd:pupilrs -2.12723 0.84740 -2.510 0.01206 ***

**Model E6: Incorporating ASD Status and VSI**

glmer(rightward ~ 1 + headrs*asd + headrs*VSIrsNoNA01 + pupilrs*asd + pupilrs*VSIrsNoNA01 + (1 + headrs + pupilrs | subnum), data = d[d$robot==1,], family='binomial', control = glmerControl(optimizer="bobyqa", optCtrl=list(maxfun=100000)));

**AIC:** 4574.8

**Fixed Effects:**

Estimate Std. Error z value Pr(>|z|)

**(Intercept) 0.7745 0.1951 3.970 7.17e-05 *****

**headrs 0.9758 0.4688 2.081 0.0374 ***

asd -0.3537 0.2894 -1.222 0.2216

VSIrsNoNA01 -0.1047 0.3015 -0.347 0.7284

**pupilrs 6.4023 0.6397 10.008 < 2e-16 *****

**headrs:asd -1.4622 0.7011 -2.086 0.0370 ***

**headrs:VSIrsNoNA01 -1.7661 0.7362 -2.399 0.0164 ***

asd:pupilrs -1.2306 0.9361 -1.315 0.1886

**VSIrsNoNA01:pupilrs 2.1326 0.9809 2.174 0.0297 ***

**Model E7: Incorporating ASD Status and VCI**

glmer(rightward ~ 1 + headrs*asd + headrs*VCIrsNoNA01 + pupilrs*asd + pupilrs*VCIrsNoNA01 + (1 + headrs + pupilrs | subnum), data = d[d$robot==1,], family='binomial', control = glmerControl(optimizer="bobyqa", optCtrl=list(maxfun=100000)));

**AIC:** 4574.6

**Fixed Effects:**

Estimate Std. Error z value Pr(>|z|)

**(Intercept) 0.75675 0.19034 3.976 7.02e-05 *****

headrs 0.63560 0.48275 1.317 0.1880

asd -0.32574 0.27550 -1.182 0.2371

VCIrsNoNA01 -0.04283 0.36907 -0.116 0.9076

**pupilrs 6.49336 0.61514 10.556 < 2e-16 *****

headrs:asd -0.78009 0.70271 -1.110 0.2670

headrs:VCIrsNoNA01 0.10768 0.94850 0.114 0.9096

asd:pupilrs -1.44844 0.87471 -1.656 0.0977 .

**VCIrsNoNA01:pupilrs 2.84591 1.16619 2.440 0.0147 ***

**Model E8: Incorporating ASD Status, Age, VSI, and VCI**

glmer(rightward ~ 1 + headrs*age + headrs*VSIrsNoNA01 + headrs*asd + headrs*VCIrsNoNA01 + pupilrs*age + pupilrs*VSIrsNoNA01 + pupilrs*asd + pupilrs*VCIrsNoNA01 + (1 + headrs + pupilrs | subnum), data = d[d$robot==1,], family='binomial', control = glmerControl(optimizer="bobyqa", optCtrl=list(maxfun=100000)));

**AIC:** 4568.2

**Fixed Effects:**

Estimate Std. Error z value Pr(>|z|)

(Intercept) 0.91930 0.61189 1.502 0.13299

headrs 1.81426 1.48218 1.224 0.22093

age -0.01342 0.05537 -0.242 0.80844

VSIrsNoNA01 -0.09417 0.31249 -0.301 0.76315

asd -0.34641 0.29054 -1.192 0.23315

VCIrsNoNA01 0.01878 0.38214 0.049 0.96081

pupilrs 0.68407 1.80639 0.379 0.70491

headrs:age -0.08327 0.13405 -0.621 0.53448

**headrs:VSIrsNoNA01 -1.95271 0.76978 -2.537 0.01119 ***

headrs:asd -1.36566 0.70790 -1.929 0.05371 .

headrs:VCIrsNoNA01 0.83518 0.94137 0.887 0.37497

**age:pupilrs 0.53138 0.16594 3.202 0.00136 ****

**VSIrsNoNA01:pupilrs 1.81593 0.91373 1.987 0.04688 ***

asd:pupilrs -0.99876 0.84575 -1.181 0.23764

**VCIrsNoNA01:pupilrs 2.16503 1.08991 1.986 0.04699 ***

**Summary of 2D Results with Covariates.** As with the 2D task, in the 3D task, among the ‘simple’ models with only one covariate, Models E5, E6, and E7, Model E5, the model with ASD and Age outperformed the models featuring VCI or VSI in place of age, by AIC score. In this model, pupil use increased with age, and decreased with ASD status. In the other two models, with VCI and with VSI, pupil use was consistently strong and increased with VCI and VSI, but there were other, additional effects (e.g., with VSI, there were effects of head usage and interactions between head usage and ASD and VSI score; with VCI, there were no effects of head usage, but there was an evident rightward judgment bias, irrespective of pupil and head cue usage). In the ‘full’ model, Model E8, incorporating ASD, Age, VSI, and VCI (each as their own main effect and in interaction with head usage and with pupil usage), head cue usage was inversely related to visuospatial ability (VSI score) and there was a trend for less head usage with ASD. In contrast, pupil usage increased with increasing age, VSI score, and VCI score. In contrast to the 2D models, the best performing model among the 3D models was the most complex model, Model E8, with the lowest AIC score.

**Overall Summary.** In both the 2D and 3D tasks considered above, simple regressions featuring age outperformed models that instead included scores of visuospatial or verbal skills, suggesting that the manner in which participants use cues to judge others’ gaze direction is not particularly strongly associated with overall metrics of either visuospatial ability or verbal ability per se, and may instead be tied to other aspects of development, here accounted for by age. However, it is important to note that the majority of observers in this study had broadly average or above average cognitive skills. Thus, our findings cannot address whether significant deficits in these skills could potentially impact gaze perception.

The results of the ‘full’ models, incorporating all covariates, confirm this (that pupil usage rises strongly with age in both 2D and 3D tasks, even when accounting for ASD status, visuospatial ability, and verbal ability; and that head usage does not follow this same pattern with age). These two models also highlight that the 2D and 3D tasks may be qualitatively different tasks, as evidenced by the different patterns of changes in cue usage in the two regressions. For example, VSI & VCI also predicted increased pupil cue usage in the 3D task, but not the 2D task; and head usage was inversely proportional to VSI score in the 3D task, but not the 2D task.

**Recoding 3D data in a head-relative reference frame**

The head and pupil direction data for the 3D condition were coded in the main manuscript as follows: head direction (-8°, 0°, and +8°), and pupil direction (-25°, -5°, +5°, +25°), *relative to the observer*. This most directly captures the direction of these cues as presented to the observer.

In actual three-dimensional heads, when the head rotates, necessarily all components of the head also rotate, including the eyes. In practice this means, for example, that pupil directions of +25° in the 3D condition resulted from three different pupil rotations, *relative to the head*: +33° in a -8° head, +25° in a 0° head, and +17° in a +8° head. We can analyze gaze direction judgments using a head-relative (not observer-relative) reference frame for pupils, though we think this is perhaps best thought of as pupil rotation and not direction. Doing so necessarily shifts some components of pupil direction information to head direction (as pupil direction in this framework is defined as the sum of pupil rotation and head direction). Regressions using this approach on 3D data result in identical AIC values (because no new information is present in the regression), but significant estimates for pupil rotation (*b* = 5.83, p < 2e-16) *and* head direction (*b* = 2.13, p = 4e-10), as would be expected. Additional exploratory analyses found no significant interactions between head direction and pupil rotation (p > 0.1), and similar patterns of the effects of age, ASD, VCI, and VSI on e.g. the use of head or pupil information.

The observer-relative reference framework was used as it is most relevant to our participants – values coded in this way reflect the physical presentation of these cues to the observer, not the inner workings of the robot, and of course our investigation focuses on aspects of the former, not the latter. The observer reference frame approach is also most consistent with the design of the 2D condition of the study, making it the clear analytic choice.

**References**

1. Harrison PL, Oakland T. ABAS-3. Torrance: Western Psychological Services; 2015.

2. Constantino JN, Gruber CP. Social responsiveness scale: SRS-2. Torrance: Western Psychological Services; 2012.

3. Chandler S, Charman T, Baird G, Simonoff E, Loucas T, Meldrum D, et al. Validation of the Social Communication Questionnaire in a population cohort of children with autism spectrum disorders. J Am Acad Child Adolesc Psychiatry. 2007;46(10):1324–32.

4. Florey J, Clifford CWG, Dakin S, Mareschal I. Spatial limitations in averaging social cues. Sci Rep . 2016;6:32210. Available from: http://www.nature.com/articles/srep32210

5. Florey J, Clifford CWG, Dakin SC, Mareschal I. Peripheral processing of gaze. J Exp Psychol Hum Percept Perform. 2015;41(4):1084–94.

6. Lovato SB, Waxman SR. Young children learning from touch screens: Taking a wider view. Front Psychol. 2016;7(JUL):1–6.
